# Bile acids supplementation modulates lipid metabolism, intestinal function, and cecal microbiota in geese

**DOI:** 10.3389/fmicb.2023.1185218

**Published:** 2023-05-26

**Authors:** Guangquan Li, Xianze Wang, Yi Liu, Shaoming Gong, Yunzhou Yang, Cui Wang, Huiying Wang, Daqian He

**Affiliations:** Institute of Animal Husbandry and Veterinary Science, Shanghai Academy of Agricultural Sciences, Shanghai, China

**Keywords:** bile acids, growth, lipid, gut, cecal microbiota

## Abstract

Bile acids(BAs) are important components of bile and play a significant role in fat metabolism. However, there is currently no systematic evaluation of the use of BAs as feed additives for geese.This study aimed to investigate the effects of adding BAs to goose feed on growth performance, lipid metabolism, intestinal morphology, mucosal barrier function, and cecal microbiota. A total of 168 28-day-old geese were randomly assigned to four treatment groups and fed diets supplemented with 0, 75, 150, or 300 mg/kg of BAs for 28 days. The addition of 75 and 150 mg/kg of BAs significantly improved the feed/gain (F/G) (*p * < 0.05).The addition of BAs decreased abdominal fat percentage and serum total cholesterol (TC) levels, with 150 mg/kg of BAs significantly reducing serum triglyceride levels and increased expression of Farnesoid X Receptor (FXR) mRNA in the liver(*p*  < 0.05), 300 mg/kg of BAs significantly increasing the expression level of liver peroxisome proliferator-activated receptor *α* (*PPARα*) (*p* < 0.05). In terms of intestinal morphology and mucosal barrier function, 150 mg/kg of BAs significantly increased villus height (VH) and VH/crypt depth (CD) in the jejunum (*p* < 0.05). The addition of 150 and 300 mg/kg of BAs significantly reduced the CD in the ileum, while increasing VH and VH/CD (*p*<0.05). Additionally, the addition of 150 and 300 mg/kg of BAs significantly increased the expression levels of zonula occludens-1 (*ZO-1*) and *occludin* in the jejunum. Simultaneously 150mg/kg and 300mg/kg BAs increased the total short-chain fatty acids (SCFA) concentrations in the jejunum and cecum(*p* < 0.05).Supplementation with BAs resulted in a significant increase in the *ɑ*-diversity of cecal microbiota and a decrease in the abundance of Proteobacteria in the cecum. The addition of 150 mg/kg of BAs significantly reduced the abundance of Bacteroidetes and increased the abundance of Firmicutes. Moreover,Linear discriminant analysis Effect Size analysis (LEfSe) showed that the abundances of bacteria producing SCFA and bile salt hydrolases (BSH) were increased in the BAs-treated group. Furthermore, Spearman’s analysis showed that the genus *Balutia*, which is negatively correlated with visceral fat area, was positively correlated with serum high-density lipoprotein cholesterol (HDL-C), while *Clostridium* was positively correlated with intestinal VH and VH/CD. In conclusion, BAs can be considered an effective feed additive for geese, as they increased SCFA concentration, improve lipid metabolism and intestinal health by enhancing the intestinal mucosal barrier, improving intestinal morphology, and altering the cecal microbiota structure.

## Introduction

In intensive poultry farming, it is common to add fat to feed to increase its energy density, improve palatability, and enhance poultry’s feed intake, thereby improving production performance (Ma et al., 2022; Saminathan et al., 2022). However, during the early stages of development, poultry have underdeveloped digestive systems, which limits their ability to digest and utilize fats (Iji et al., 2001; Vertiprakhov et al., 2022). When they consume more energy than they need, it can lead to increased fat accumulation in their abdominal area, as well as health problems such as fatty liver (Sanz, 1999; Crespo and Esteve-Garcia, 2001). This places a significant burden on their liver and intestines. Therefore, it is feasible to develop additives that can help poultry digest fats while ensuring their production performance. For example, adding probiotics or prebiotics can improve gut health and enhance the digestibility of fats, while antibiotics or antibiotic alternatives can also be used as growth promoters to improve poultry digestive performance (Duskaev et al., 2021; Wang et al., 2022).

Bile acids, which are the primary component of bile, are synthesized by the liver and stored in the gallbladder (Hofmann and Hagey, 2008). As a fat emulsifier, BAs effectively emulsify lipids into minute chylomicron particles, which augment lipid metabolism by promoting contact with lipases (Lefebvre et al., 2009). Furthermore, BAs act as signaling molecules that bind to the farnesoid X receptor (FXR), which plays a role in regulating lipid metabolism (Arthur et al., 2019; Li et al., 2019). These mechanisms promote the absorption of lipids and fat-soluble vitamins, while also mitigating the negative impact of a high-fat diet on lipid deposition (Saeed et al., 2017; Adachi, 2018), resulting in improved growth performance (Watanabe et al., 2006). Additionally, BAs have been found to play a critical role in physiological functions such as inflammation and energy homeostasis (Reschly et al., 2008; Wu et al., 2019). Recent studies have emphasized the importance of studying how BAs and the gut microbiota of poultry interact with each other. BAs have a significant impact on the composition and ratio of intestinal microbiota, which can affect their metabolism and ultimately influence the metabolism, immune function, and overall health of the host. This interaction between BAs and intestinal microbiota is a crucial factor that influences the health of the host (Liu et al., 2022a,b). Additionally, the intestinal microbiota can produce beneficial secondary metabolites, such as BSH, through the metabolism of BAs, which can further contribute to the health of the host (Burns et al., 2021). There is evidence to suggest that BAs can stimulate the growth of beneficial bacteria and hinder the growth of harmful bacteria in the gut, which may reduce the risk of enteric infections (Inagaki et al., 2006; Gao et al., 2022). Research on the impact of BAS on intestinal microbiota can help to understand the interaction mechanism between intestinal microbiota and the host, and provide important theoretical support for the development of new health management methods and disease treatment strategies.

In 2014, BAs were granted approval as a feed additive and can now be utilized in broiler chickens, weaned piglets, and freshwater fish. However, no research has been conducted on the effectiveness and safety of adding BAs to geese.

## Materials and methods

### Animal ethics

Animal handling and treatment were carried out in accordance with the Chinese Animal Welfare Guidelines. This research was approved by the Laboratory Animal Ethics Committee of the Shanghai Academy of Agricultural Sciences (SAASPZ0522046).

### Animals and experimental design

BAs used in this experiment were purchased from Zhengzhou Shangshui Biotechnology Co., Ltd., Zhengzhou City, Henan Province, China. with a purity of 99.5%. The BAs used in this experiment were synthesized artificially and the content of each component in the BAs was detected using liquid chromatography–tandem mass spectrometry. The detection results showed that the BAs contained 15.00% of cholanic acid, 44.00% of hyodesoxycholic acid, and 40.50% of chenodeoxycholic acid. A total of 168 28-day-old male Holldobagy geese were selected for the experiment, which were purchased from Xiangtian Ge Family Farm in He County, Ma’anshan City, Anhui Province, China. All geese were randomly divided into four groups (with dietary BAs concentrations of 0, 75, 150, and 300 mg/kg), with six replicates per group and 7 geese per replicate. The diet used in this study was formulated to meet the nutrient requirements of geese in China, based on the recommendations of NRC 1994 (Table 1). All geese were raised in a closed house with a density of 0.5m^2^ per goose, with temperature maintained at around 15°C. They were exposed to natural light during the day and provided with artificial light at night. Throughout the experiment, the geese had unrestricted access to feed or water, and their health status and vaccination were monitored regularly.

**Table 1:** Composition and nutrient level of experiment diets (air-dry basis).

Items	Content (%)
Ingredients	
Corn	67.92
Soybean meal	24.90
HSW	0.00
Soybean oil	2.00
Lys	0.09
Met	0.09
Thr	0.00
Premix^a^	5.00
Total	100.00
Nutrient level	
CP	16.00
ME (MJ/kg)^b^	12.40
*CF*	2.56
Ca	0.79
P	0.51
Lys	0.90
Met+Cys	0.66
Thr	0.63

^a^One kilogram of the premix contained the following: Fe 100 mg, Cu 8 mg, Mn 120 mg, Zn 100 mg, Se 0.4 mg, Co 1.0 mg, I 0.4 mg, VA 8330 IU, VB1 2.0 mg, VB2.8 mg,VB6 1.2 mg, VB12 0.03 mg, VD31440 IU, VE 30 IU, biotin 0.2 mg, folic acid 2.0 mg, pantothenic acid 20 mg, niacin acid 40 mg. ^b^Nutrient levels were all calculated values.

### Growth performance

During the experimental period, daily feed intake of the geese in each treatment group was carefully recorded. In addition, the geese were subjected to a 10-h fast and a 2-h water withholding period before their morning feeding at 35, 49, and 63 days of age, followed by an accurate weight measurement to determine their body weight (BW) on an empty stomach. These measures were conducted to calculate the average daily gain (ADG), average daily feed intake (ADFI), and F/G at the ages of 35–49, 49–63, and 35–63 days, respectively.

ADG = (weight at time 2 - weight at time 1)/(number of days between time 1 and time 2)

ADFI = total feed intake/number of days× test geese

F/G = total feed intake/total weight gain

### Serum lipid

At the conclusion of the study, a single goose was chosen from each trial whose body weight closely approximated the replicate average. Blood collection of 4 mL was taken from the vein of each chosen goose. Subsequently, the blood samples were left at 37°C for 4 h and centrifuged at 4000 rpm to separate the serum. The serum samples were analyzed for levels of total cholesterol (TC), triglycerides (TG), HDL-C, and low-density lipoprotein cholesterol (LDL-C) using an automated biochemical analyzer (Mindray BS200, China).

### Liver lipids and gut mucosal barrier

For each replicate (*n* = 7), a goose with a weight close to the mean was selected and euthanized by bleeding from the neck with efforts made to minimize the suffering of the geese during the procedure. All samples were obtained from 24 geese. The liver was completely removed and all abdominal fat was peeled off, weighed to calculate the organ index (organ weight, g/body weight, kg). A small quantity of liver and jejunal mucosa were collected and stored in a 2 ml cryotube, which was then placed in liquid nitrogen for preservation.

The liver and jejunal mucosa RNA was extracted using Trizol (Invitrogen, Carlsbad, CA, USA), and a reverse transcription kit (TransScript, TransGen, Beijing, China) was used to transcribe the total RNA with a concentration and purity measured using Nano-drop 1,000 Spectrophotometer (Thermo Fisher Scientific, Waltham, MA, USA) into cDNA. The *β*-actin gene was selected as a reference gene. Real-time PCR was conducted to determine the expression levels of target genes using the TransGen TB Green kit (TransGen Bio Inc., Beijing, China) and a fluorescence quantitative reaction instrument (QuantStudio 5, Thermo Fisher Scientific, MA, USA), including sterol-regulatory element binding proteins (*SREBP-1*), *PPARα*, fatty acid synthase (*FAS*), Acetyl CoA carboxylase (*ACCA*), Farnesoid X Receptor (*FXR*), zona occludens 1 (*ZO-1*), *Occludin* and *Claudin-1* with primer sequences provided in Table 2, the *β*-actin gene was a reference gene. For data analysis, a relative quantitative method (2^-^ΔΔCT^) was used.

**Table 2:** Primer sequences for genes used in RT-qPCR.

Genes	Primer sequence 5′-3´	Genbank
ZO-1	F:CTAGCTAGCGTACAGTACAC	XM_013177404.1
	R:CTCTCTCATAGGCAGGAAAC	
Occludin	F:GCTGGGCTACAACTACGGGT	XM_013199669
	R:ACGATGGAGGCGATGAGC	
Claudin-1	F:GGAAGATGACCAGGTGAAG	XM_013199194.1
	R:GGAAGATGACCAGGTGAAG	
SREBP-1	F:CCGCTCATCCATCAACGAC	EU_333990
	R:GGCTGAGGTTCTCCTGCTTC	
PPARα	F:CCACAGCTCCAGGTAGCATAG	AF_481797
	R:AGGCACTTTTGAAAACGACAG	
FAS	F:ATCTCTGGCACGGTCTCTTG	EU_770327
	R:GCTGCACTGATCCTTTG	
ACCA	F:CCGGGAGGTTAATGGAAGGAC	EF_990142
	R:TGTGCCCTCAGCACTCTTG	
FXR	F:TTTGCTCCAGCTGGACTCAG	XM_048071150.1
	R:AGAAAGAGACGGTAGTTCCAGAG	
β-actin	F:TCCGTGACATCAAGGAGAAG	XM_013174886.1
	R:CATGATGGAGTTGAAGGTGG	

### Intestinal morphology

Jejunum and ileum intestinal segments were collected and immediately placed on ice. The segments were washed with 4°C pre-cooled PBS for 20 s to remove intestinal contents. Three-centimeter long complete middle segments were collected, fixed in 4% paraformaldehyde solution (Aladdin, Shanghai, China) for 24 h, and then subjected to treatments such as water washing, dehydration, transparency, paraffin embedding (Electron Microscopy Sciences, Hatfield, USA), and sectioning using Leica RM2016 microtome (Leica, Germany). After staining with hematoxylin and eosin, the sections were observed under an optical microscope (Olympus BX53, Olympus Optical Co. Ltd., Shenzhen, China) to examine the morphological parameters of the jejunum, and ileum. CD, VH and the VH/CD were calculated after morphological measurements.

### Cecal microbiota

Approximately 1 g of cecal chyme was collected and transferred into sterile cryovials, stored in liquid nitrogen, and transferred to a −80°C ultra-low temperature freezer after sampling. DNA was extracted from cecal contents using a DNA extraction kit. The purity and quality of DNA were determined by 1.0% agarose gel electrophoresis. The V3-V4 region of the 16S rDNA was amplified using specific primers (338F, 5′-ACTCCTACGGGAGGCAGCA-3′, 806R: 5′-GGACTACHVGGGTWTCTAAT-3′). The PCR products were purified and recovered after being analyzed by lipopolysaccharide agarose gel electrophoresis. MiSeq library was constructed and Illumina sequencing was performed by Shanghai Personal Biotechnology Co., Ltd. (Shanghai, China).

### Intestine short-chain fatty acids

Aseptically collect 1 g of chyme from the cecum and colon, and add an appropriate amount of PBS (pH = 7.4) to a 2 mL Eppendorf tube. Quickly freeze and store the sample in liquid nitrogen. After thawing the chyme, homogenize it thoroughly using a homogenizer. Centrifuge the homogenate for 20 min at 3000 rpm, and collect the supernatant. The concentration of total SCFA in the cecum and colon samples of each group was detected using an Infinite F50 microplate reader (Tecan, Switzerland) and an enzyme-linked immunosorbent assay (ELISA) kit (Jiangsu Jingmei Biotechnology Co., Ltd., Yancheng, China) for goose SCFA.

### Statistical analysis

Use Microsoft Excel 2007 to organize various data. Perform one-way ANOVA analysis and Duncan multiple comparison of variance using SPSS 25.0 software (IBM, New York, USA). Polynomial contrasts were used to determine the linear and quadratic effects of dietary BAs supplementation levels. Results will be presented as mean ± standard deviation and significant differences will be indicated as *p* < 0.05.

Raw cecal microbiota sequencing data were quality-controlled using QIIME 2.0 software. Optimized sequences were obtained through sequence splicing, screening, and elimination of chimeras. USEARCH 7.0 software was used for cluster analysis of amplicon sequence variants (ASV). Greengenes data were used for the taxonomic analysis of ASV sequences, and the community composition of each sample was counted at the levels of phylum, class, order, family, genus, and species. Principal coordinate analysis (PCoA) of the cecal microbiota was performed using R language, and data visualization was conducted using STAMP 2.1.3 software. ɑ-diversity indices were calculated using Mothur 1.2 software. Using Phylogenetic Investigation of Communities by Reconstruction of Unobserved States (PICRUSt2) to predict the metabolic capabilities of the cecal microbiota.

## Results

### Growth performance

During the experiment, the geese remained in good health, and there were no eliminations or deaths due to stress or disease. The growth performance during the experimental period is presented in Table 3. No significant differences (*p* > 0.05) in BW were observed among the groups at 35, 49, and 63d. No significant differences (*p* > 0.05) were found in ADFI among the groups at 35–49, 49–63, and 35–63d. Regarding ADG, there were no significant differences (*p* > 0.05) among the groups at 49–63 and 35–63d. However, the addition of 150 mg/kg BAs increased ADG by 12.16% (*p* < 0.05) at 35–49d. In terms of F/G, the addition of 75, 150, and 300 mg/kg significantly increased F/G at 35–49d (*p* < 0.05). No significant differences (*p* > 0.05) were found among the groups at 49–63d. The addition of 75 and 150 mg/kg significantly improved F/G at 35–63d (*p* < 0.05).

**Table 3:** The effects of supplemental BAs on the growth performance of geese^a^.

Items	Treatment				*p*-value		
	0	75 mg/kg	150 mg/kg	300 mg/kg	ANOVA	Linear	Quadratic
BW, g							
28d	1165.26 ± 206.95	1170.98 ± 154.14	1166.33 ± 203.65	1166.67 ± 238.62	0.999	0.904	0.991
35d	1770.24 ± 189.73	1762.52 ± 174.93	1762.21 ± 219.64	1754.86 ± 206.08	0.988	0.823	0.944
49d	2998.29 ± 316.94	3063.50 ± 319.07	3139.62 ± 370.18	3068.14 ± 319.41	0.286	0.590	0.317
63d	3878.79 ± 348.67	3982.95 ± 435.42	4011.40 ± 426.14	3929.00 ± 362.53	0.427	0.400	0.571
ADFI, g/d							
28–35d	293.64 ± 1.49	294.86 ± 1.93	294.8 ± 2.12	293.71 ± 0.76	0.424	0.117	0.302
35–49d	305.55 ± 4.63	300.09 ± 2.60	306.80 ± 7.86	306.18 ± 3.50	0.116	0.088	0.052
49–63d	364.27 ± 6.35	366.43 ± 7.03	359.58 ± 12.48	354.23 ± 8.91	0.124	0.950	0.097
35–63d	334.91 ± 3.66	333.26 ± 3.63	333.19 ± 8.42	330.21 ± 5.70	0.559	0.450	0.447
ADG, g							
28–35d	86.43 ± 8.40	86.74 ± 7.43	85.78 ± 8.95	85.08 ± 8.31	0.802	0.773	0.637
35–49d	87.72 ± 15.72^b^	92.93 ± 16.85^ab^	98.39 ± 17.44^a^	93.81 ± 14.97^ab^	0.031	0.335	0.048
49–63d	62.89 ± 12.31	65.68 ± 14.75	62.28 ± 11.41	61.49 ± 13.34	0.484	0.401	0.345
35–63d	75.31 ± 10.18	79.30 ± 13.77	80.34 ± 12.13	77.65 ± 11.16	0.235	0.260	0.349
F/G							
28–35d	3.40 ± 0.13	3.40 ± 0.11	3.44 ± 0.15	3.46 ± 0.11	0.837	0.956	0.657
35–49d	3.48 ± 0.26^a^	3.23 ± 0.10^b^	3.12 ± 0.12^b^	3.27 ± 0.15^b^	0.010	0.108	0.034
49–63d	5.80 ± 0.27	5.62 ± 0.53	5.79 ± 0.36	5.78 ± 0.41	0.837	0.430	0.658
35–63d	4.47 ± 0.26^a^	4.21 ± 0.21^b^	4.15 ± 0.11^b^	4.25 ± 0.14^ab^	0.046	0.088	0.063

^a^Different lowercase letters with the same column date meant significant difference (*P* < 0.05).

### Bile acid receptor expression

**Figure 1:**
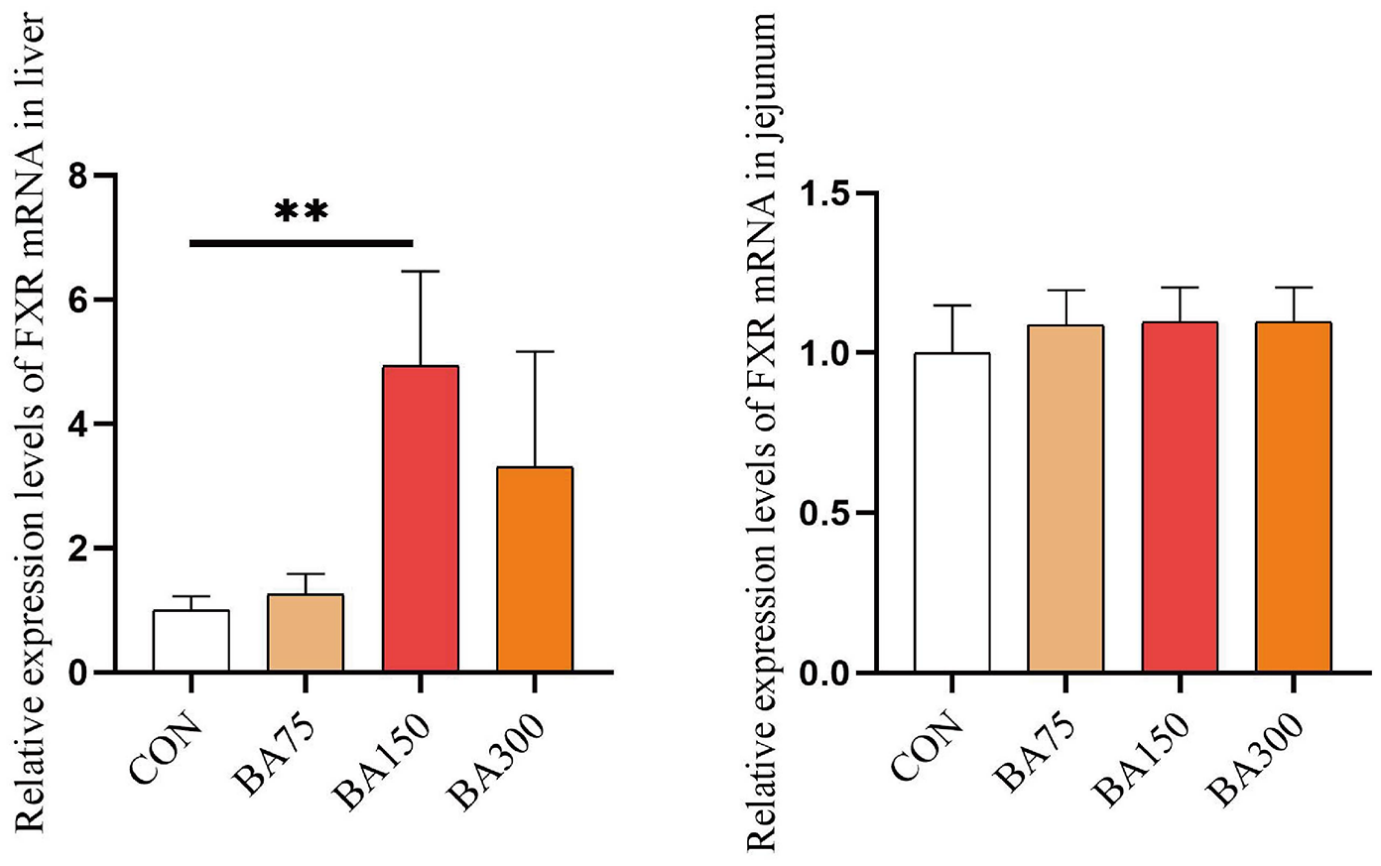
Effects of different levels of BAs on the expression of goose bile acid receptor. FXR, Farnesoid X Receptor. CON, 0 mg/kg BAs; BA75, 75 mg/kg BAs; BA150, 150 mg/kg BAs; BA300, 300 mg/kg BAs. *p < 0.05, **p < 0.01.

We examined the expression of bile acid receptor-FXR mRNA in both the liver and jejunum. According to the results presented in Figure 1, the addition of bile acid to the diet led to an increase in the expression of FXR in the liver. Specifically, both the 150 mg/kg significantly elevated the levels of FXR mRNA expression in the liver (*p* < 0.05), whereas there were no significant differences among the groups in the jejunum. Furthermore, the expression of FXR mRNA in the liver was found to be higher than that in the jejunum, suggesting that FXR may play a more crucial role or have a broader range of physiological effects in the liver.

### Lipid metabolism

**Figure 2:**
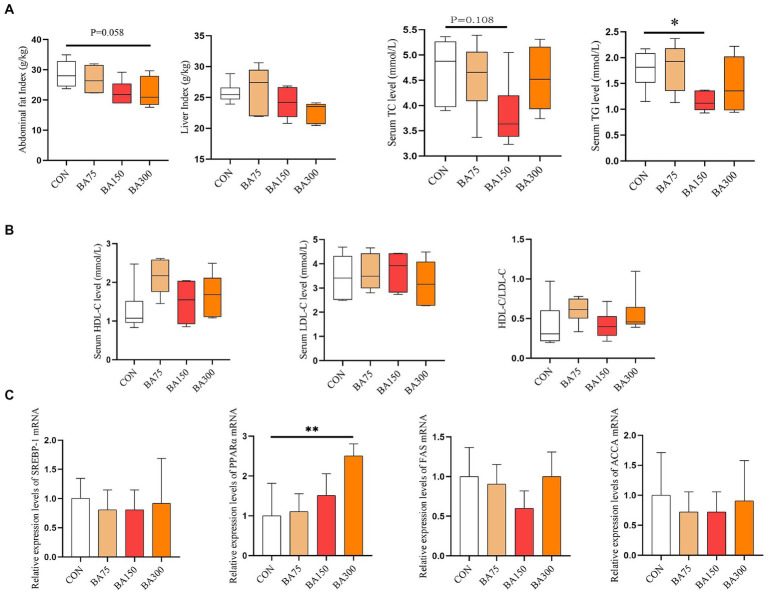
Effects of different levels of BAs on fat metabolism in geese. **(A)** Effects of BAs on liver index and visceral fat index. **(B)** Effects of BAs on serum total cholesterol (TC), triglycerides (TG), high-density lipoprotein cholesterol (HDL-C), and low-density lipoprotein cholesterol (LDL-C). **(C)** Effects of BAs on mRNA expression of liver lipid metabolism genes, sterol-regulatory element binding proteins (*SREBP-1*), peroxisome proliferators-activated receptor α (*PPARα*), fatty acid synthase (*FAS*), Acetyl CoA carboxylase (*ACCA*). *p < 0.05, **p < 0.01.

In order to investigate the impact of different levels of BAs on the metabolism of goose fat, we first examined their effects on liver and abdominal fat indices (Figure 2A). We found no significant differences in liver index between the groups (*p* > 0.05). As the level of BAs increased, the abdominal fat index gradually decreased. At 300 mg/kg, there was a trend toward lower abdominal fat (*p* = 0.058).

We determined the differences in serum lipid metabolism among different treatment groups at the same time. As shown in Figure 2B, the addition of 75 mg/kg of BAs had no significant effect on serum TG levels (*p* > 0.05), while the addition of 150 mg/kg of BAs significantly reduced the levels (*p* < 0.05). As the level of dietary BAs increased, HDL-C and HDL-C/LDL-C levels also increased, but there was no statistically significant difference among the groups (*p* > 0.05).

To further investigate its impact on lipid metabolism, we measured the mRNA expression levels of liver *SREBP-1*, *PPARα*, *FAS,* and *ACCA* (Figure 2C). The addition of 75, 150, and 300/kg decreased the expression of *SREBP-1*, *FAS,* and *ACCA* on the liver, but there was no significant difference among the groups (*p* > 0.05). However, adding BAs increased the expression levels of *PPARα*, and at a level of 300 mg/kg, *PPARα* reached a significant difference (*p* < 0.05).

### Intestinal morphology and mucosal barrier

**Figure 3:**
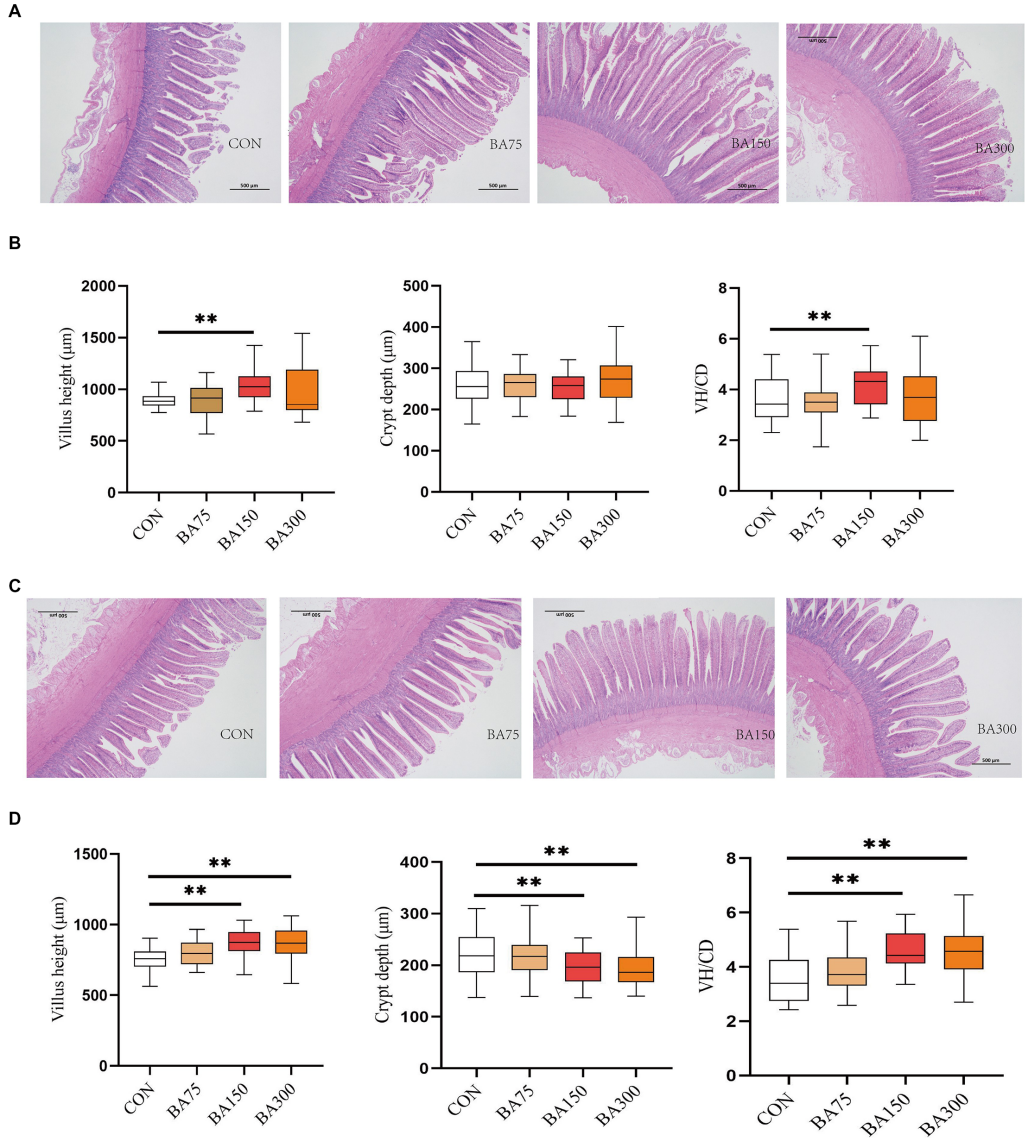
Effects of different levels of BAs on the morphology of the jejunum and ileum in geese. **(A)** Effects of BAs on the morphology of the jejunum after Hematoxylin and Erosion staining. Scale bar, 500 μm. **(B)** Villus height (VH), crypt depth (CD), and VH/CD ratio in the jejunum of geese fed a basal diet with different levels of BAs. **(C)** Effects of BAs on the morphology of the ileum after Hematoxylin and Erosion staining. Scale bar, 500 μm. **(D)** Villus height (VH), crypt depth (CD), and VH/CD ratio in the ileum of geese fed a basal diet with different levels of BAs. *p < 0.05, **p < 0.01.

Figure 3A shows the changes in the morphology of the cecum following the addition of 0, 75, 150, and 300 mg/kg of dietary BAs. We found that the addition of BAs to the diet did not have a significant effect on CD in jejunum (*p* > 0.05), but 150 mg/kg of BAs significantly increased VH and VH/CD (*p* < 0.05) (Figure 3B). Figure 3C shows the changes in the morphology of the ileum in each treatment group. Unlike jejunum, the addition of 150 and 300 mg/kg significantly decreased CD in the ileum, increased VH and VH/CD (*p* < 0.05) (Figure 3D).

**Figure 4:**
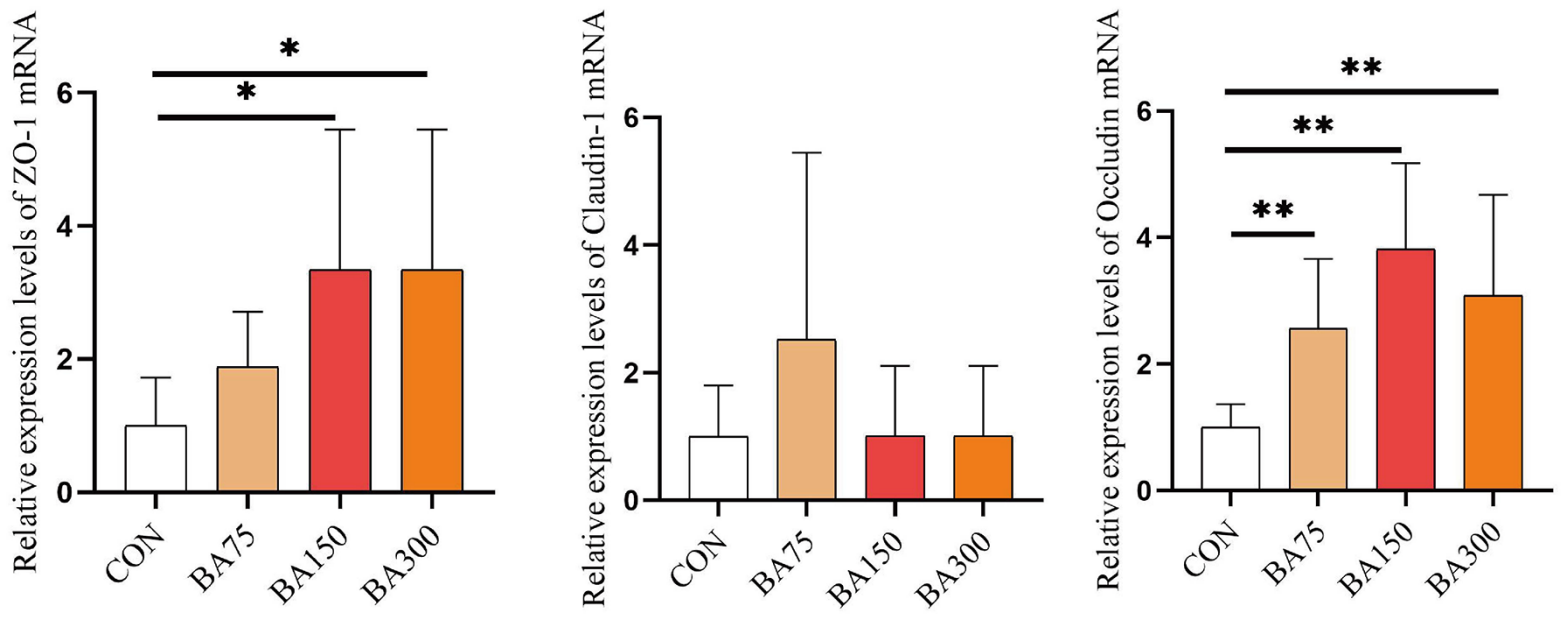
Effects of different levels of BAs on the integrity of the jejunal barrier in geese. Effects of BAs on jejunal expression of zona occludens 1 (*ZO-1*), *Occludin*, and *Claudin-1* mRNA in geese. *p < 0.05, **p < 0.01.

To further investigate the impact of BAs on intestinal barrier integrity, we measured the relative expression levels of *Occludin*, *ZO-1* and *Claudin-1* mRNA in the jejunum (Figure 4). Our findings revealed that the addition of BAs increased the expression levels of all three genes. Notably, the expression of *ZO-1* was significantly increased with the addition of 150 mg and 300 mg/kg of BAs (*p* < 0.05), with the highest expression observed with the addition of 150 mg/kg. Although there was no significant difference in *Claudin-1* expression levels among the groups (*p* > 0.05), the expression of *Occludin* was increased with the addition of 75, 150, and 300 mg/kg of BAs (*p* < 0.05).

### Cecal microbiota diversity

**Figure 5:**
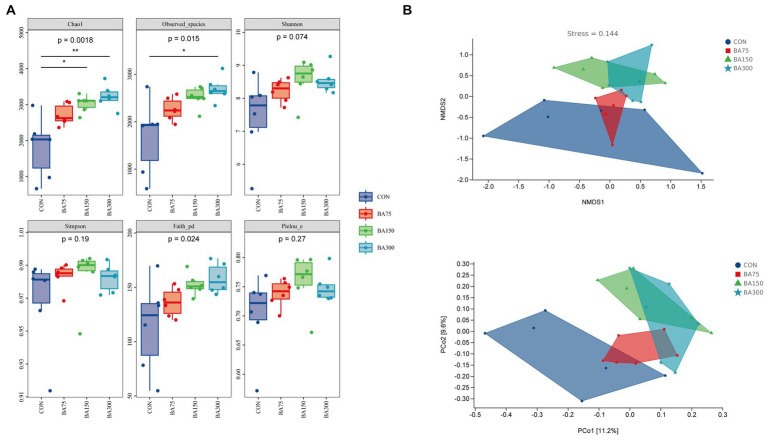
Effects of different levels of BAs on the diversity of cecum microbiota in geese. **(A)** Effects of adding BAs to the basal diet on the *ɑ*-diversity of fecal bacteria in geese. Boxplots show the differences in Chao1, Observed species, Shannon, Simpson, Faith’s PD, and Pielou’s evenness indices between groups. **(B)** The *β*-diversity analysis of cecum microbiota among groups. PCoA and NMDS analyses were performed based on Bray–Curtis distance, and different colors and shapes represent samples from different groups. *p < 0.05, **p < 0.01.

To evaluate the effect of BAs on the microbiota in the cecum of geese, we analyzed the *α*-diversity and *β*-diversity between groups based on sequencing data. In terms of *α*-diversity (Figure 5A), we measured the indices of Chao1, Observed species, Shannon, Simpson, Faith’s PD, and Pielou’s evenness, where Chao1 and Observed species represent richness, Shannon and Simpson represent diversity, Faith’s PD represents evolutionary diversity, and Pielou’s evenness represents evenness. The results showed no significant differences in Shannon, Simpson, and Pielou’s indices between groups (*p* > 0.05). However, adding 150 mg/kg of BAs significantly increased Chao1 (*p* < 0.05), and adding 300 mg/kg of BAs significantly increased Chao1, Observed species, and Faith’s PD indices (*p* < 0.05). The *β*-diversity is showed in Figure 5B. The PCoA based on Bray-Curtis distance showed the *β*-diversity between groups. We found that there was some overlap between the samples treated with 0 mg/kg and 75 mg/kg of BAs, while those treated with 150 mg/kg and 300 mg/kg of BAs showed a more obvious clustering, although the difference was not significant. Therefore, further analysis is needed to explore the differences in microbial composition between groups.

### Microbial composition

**Figure 6:**
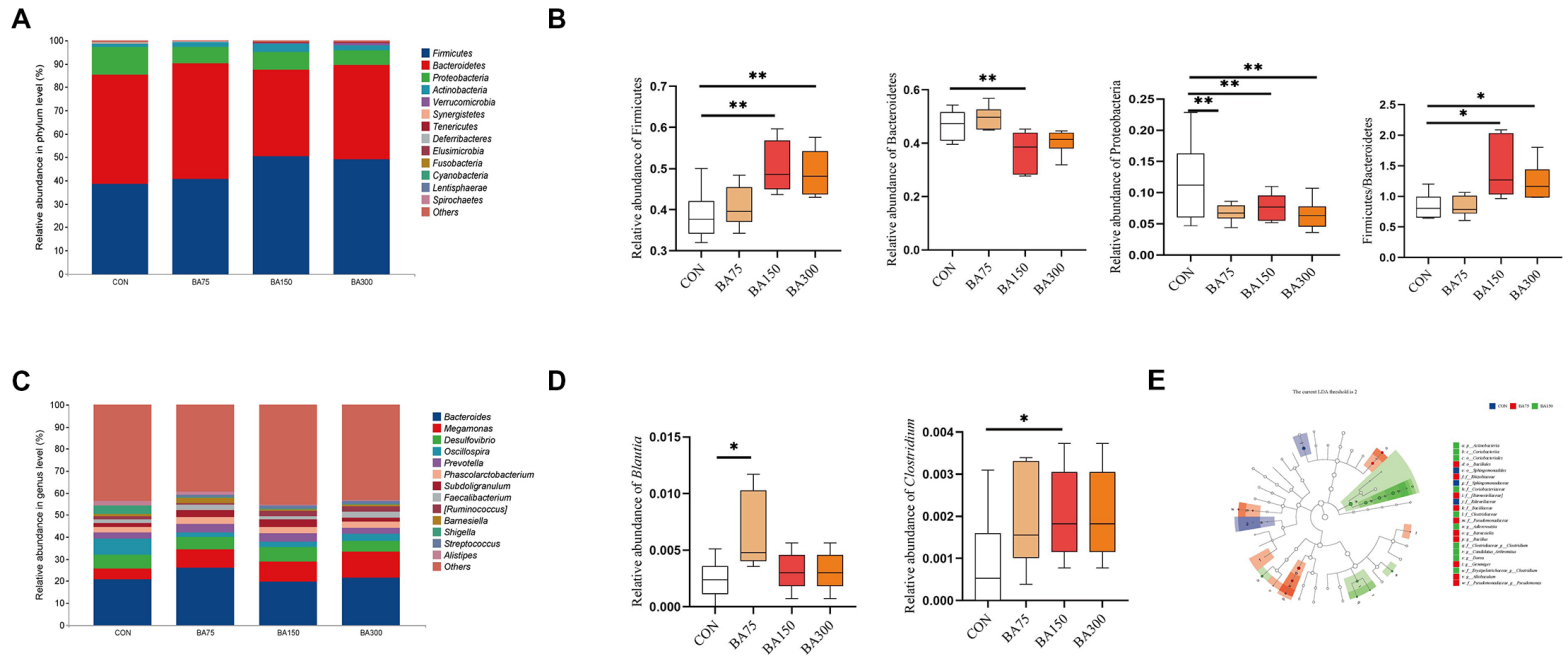
Effects of different BAs on changes in fecal microbiota of geese. At the phylum **(A)** and genus **(B)** levels, the classification composition and relative abundance of fecal microbiota in each group. The abundance of Firmicutes, Bacteroidetes, and Proteobacteria and the ratio of Firmicutes to Bacteroidetes at the phylum level **(C)**. The relative abundance of Blautia and Clostridium at the genus level **(D)**. Linear discriminant analysis (LDA) (LDA > 2.0, *p* < 0.05) of cecal microbiomes **(E)**. *p < 0.05, **p < 0.01.

To investigate the changes in gut bacteria abundance among different groups, we calculated the impact of different levels of BAs on the composition of cecal microbiota in geese. Subsequently, we constructed a bar graph to represent the relative abundance of cecal bacteria in each sample. To further elucidate the abundance levels and proportions of different phyla within each sample, we generated a column accumulation chart based on the relative abundance of bacteria (Figure 6A).

The primary microbial phyla in the cecum are Firmicutes, Bacteroides, Proteobacteria, and Actinobacteria, which together account for over 98% of total abundance in each group with different dosages of BAs supplementation—namely 98.46% for 0 mg/kg, 99.19% for 75 mg/kg, 98.45% for 150 mg/kg, and 97.81% for 300 mg/kg. Notably, the phylum Bacteroidetes exhibited the highest relative abundance in samples supplemented with 0 mg/kg and 75 mg/kg, reaching 46.83 and 49.60%, respectively. By contrast, the addition of 150 mg/kg caused a 26.50% decrease in the abundance of this phylum (*p* < 0.05) (Figure 6C). In contrast, the highest abundance of Firmicutes was observed in samples supplemented with 150 mg/kg and 300 mg/kg, with relative abundances of 50.28 and 48.97%, respectively, representing increases of 30.29 and 26.90% compared with 0 mg/kg (*p* < 0.05). In addition, the Firmicutes to Bacteroidetes (F/B) ratio increased significantly with the addition of 150 and 300 mg/kg BAs (*p* < 0.05). Moreover, there was a significant decrease in Proteobacteria abundance with the supplementation of 75, 150, and 300 mg/kg BAs (*p* < 0.05). The dominant microbial genera in the cecum were found to be *Bacteroides, Megamonas, Desulfovibrio,* and *Oscillospira*, which together accounted for 39.41, 43.33, 37.84, and 41.48%, respectively (Figures 6B). One-way ANOVA analysis of the top 50 abundant genera in each sample showed significant increases in the abundance of *Blautia* with the addition of 75 mg/kg BAs and the abundance of with 150 mg/kg BAs (*p* < 0.05) (Figure 6D). Using Linear Discriminant Analysis Effect Size (LEfSe) analysis, biomarkers for each group were identified, including *f__Rikenellaceae, f__Sphingomonadaceae* and *o_Sphingomonadales* for the group that received 0 mg/kg BAs, *f_[Barnesiellaceae], g_Barnesiella, f_Rhizobiaceae, g_Allobaculum, o_Bacillales, g_Gemmiger, f_Bacillaceae, g__Bacillus, f_Pseudomonadaceae*, and *f_Pseudomonadaceae_g__Pseudomonas* for the group that received 75 mg/kg BAs, and *p_Actinobacteria, o_Coriobacteriales, f__Coriobacteriaceae, c_Coriobacteriia*, *f__Erysipelotrichaceae_g__Clostridium, f__Clostridiaceae, f_Clostridiaceae_g__Clostridium, g__Dorea, g_Adlercreutzia*, and *g_Adlercreutzia Candidatus Arthromitus* for the group that received 150 mg/kg BAs (Figure 6E).

### Intestine short-chain fatty acids

**Figure 7:**
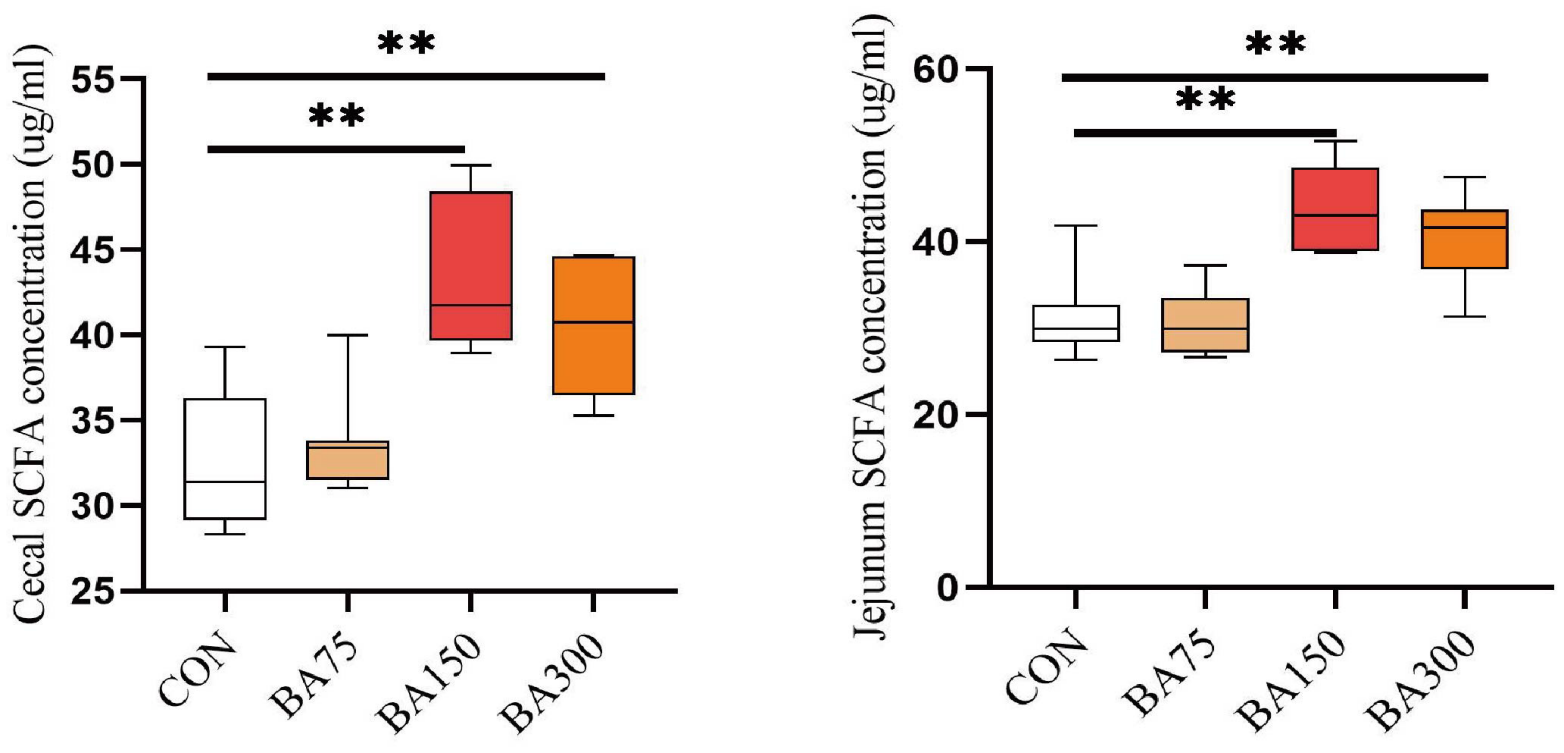
Effects of different levels of BAs on the content of SCFA in goose jejunum and cecum. SCFA, short chain fatty acids. *p < 0.05, **p < 0.01.

We found an increase in the abundance of SCFA-producing bacteria in the cecum by 16S sequencing. Therefore, we detected the total concentration of SCFA in the jejunum and cecum using ELISA. As shown in Figure 7, we observed that the addition of 75 mg/kg of BAs had no significant effect on the SCFA concentration in the jejunum and ileum (*p* > 0.05), but the addition of 150 mg/kg and 300 mg/kg of bile acid significantly increased the SCFA concentration in the cecum and jejunum (*p* < 0.05).

### Function prediction

**Figure 8:**
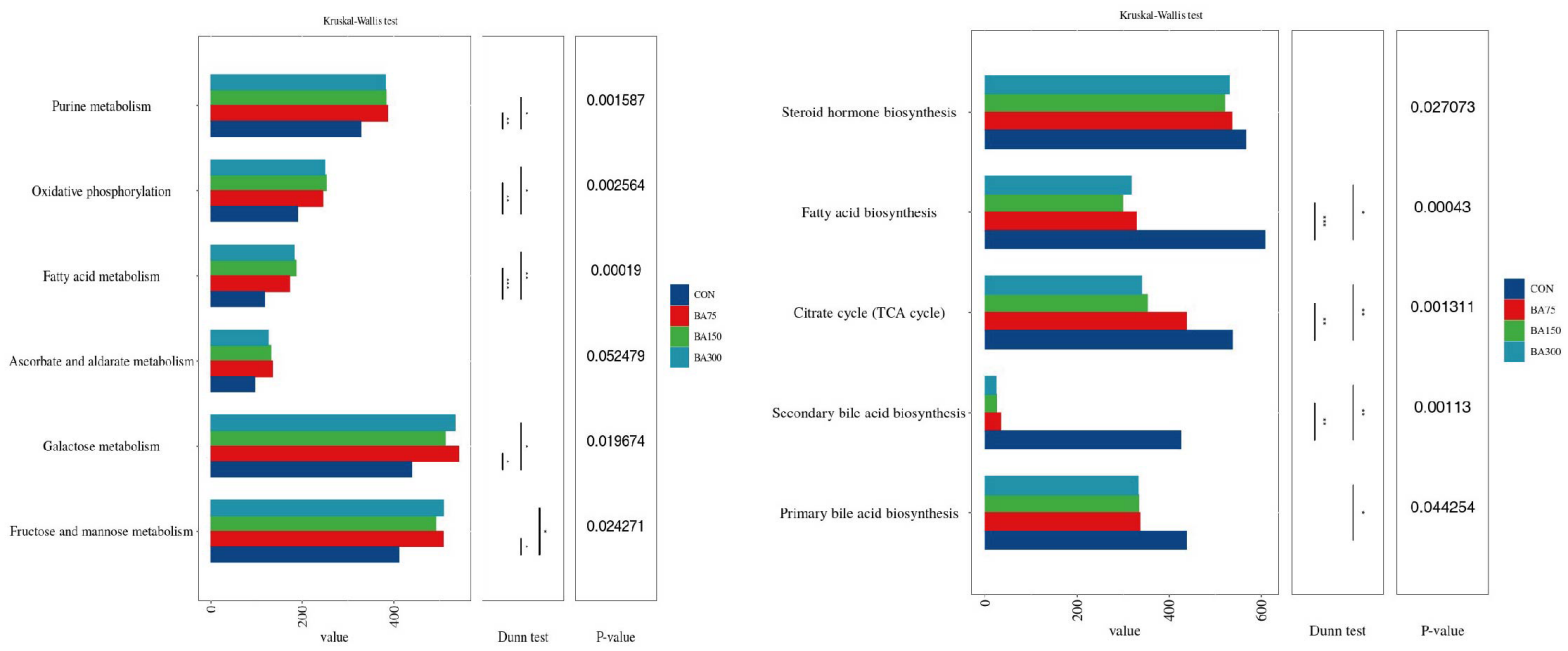
Effect of different levels of BAs on the KEGG pathways of cecal microbiota in geese, and *p < 0.05 and, **p <0.01 or ***p <0.001 indicate significant correlations. The *Y*-axis represents metabolic pathways, and the *X*-axis represents the relative abundance under each pathway.

We utilized PICRUSt2 to predict the KEGG pathways associated with the cecal microbiota involved in lipid metabolism (Figure 8). The results showed that the abundance of certain metabolic pathways was increased with 75 mg/kg of BAs, including Fructose and mannose metabolism, Purine metabolism, and Galactose metabolism (*p* < 0.05). Fatty acid metabolism and Oxidative phosphorylation were increased with 150 and 300 mg/kg of BAs (*p* < 0.05). In contrast, Secondary bile acid biosynthesis, TCA cycle, and Fatty acid biosynthesis were decreased with 150 and 300 mg/kg of BAs, and Primary bile acid biosynthesis was decreased with 300 mg/kg of BAs (*p* < 0.05). However, 75 mg/kg of BAs had no significant effect on the above pathways (*p* > 0.05).

### Main parameters and cecum microbiota

**Figure 9:**
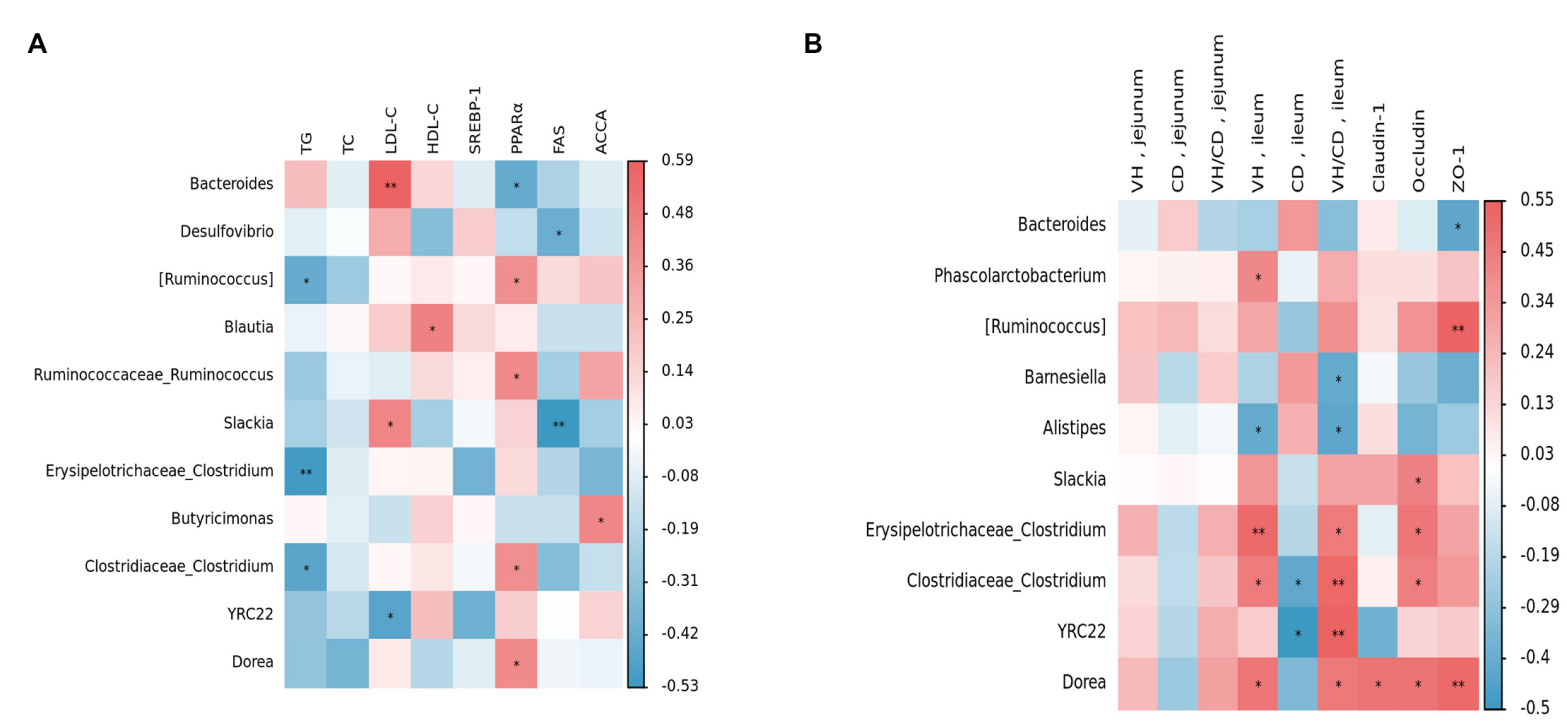
Relationships between main parameters and the top 30 genera in the cecum. Red represents positive correlation, blue represents negative correlation, and *p < 0.05 and **p < 0.01 represent significant correlations. **(A)** Relationships between fat metabolism and gut microbiota. **(B)** Relationships between gut morphology and gut microbiota.

In order to gain a deeper understanding of the relationship between the cecal microbiota and lipid metabolism and intestinal function, the Spearman correlation analysis was conducted based on the relative abundance of the top 30 bacterial genera’s main species. This was done to determine the relationships between them. Figure 9A presents the relationship between lipid metabolism and genera. The findings revealed that *[Ruminococcus], Erysipelotrichaceae Clostridium,* and *Clostridiaceae Clostridium* were negatively correlated with TG levels (*p* < 0.05), whereas *Bacteroides, Slackia*, and LDL-C levels were positively correlated (*p* < 0.05). Additionally, *Blautia* was positively associated with HDL-C levels (*p* < 0.05). Regarding liver lipid metabolism, *Bacteroides* was found to be negatively correlated with the expression level of liver *PPARα* mRNA (*p* < 0.05). In contrast, *[Ruminococcus], Ruminococcaceae Ruminococcus, Clostridiaceae Clostridium*, and *Dorea* were positively correlated with *PPARα* expression level (*p* < 0.05). Similarly, *Desulfovibrio,* and *Slackia* exhibited a negative correlation with *FAS* expression level (*p* < 0.05).

Figure 9B shows the relationship between bacterial genera, VH, CD, and mucosal barriers. The results indicated that there were no significant differences between the parameters of the jejunum and the genera. In the ileum, *Phascolarctobacterium, Erysipelotrichaceae Clostridium, Clostridiaceae Clostridium,* and *Dorea* were positively correlated with VH (*p* < 0.05). Additionally, *Clostridiaceae Clostridium* and *YRC22* were negatively correlated with CD (*p* < 0.05). Furthermore, *Barnesiella* and *Alistipes* exhibited a negative correlation with VH/CD (*p* < 0.05). In terms of gene expression related to intestinal mucosal barriers, the results showed that *Slackia, Erysipelotrichaceae Clostridium, Clostridiaceae Clostridium, Dorea*, and *Occludin* were positively correlated (*p* < 0.05). Conversely, *Bacteroides* was found to be negatively correlated with the expression of *ZO-1* (*p* < 0.05) but positively correlated with *[Ruminococcus]* and *Dorea* expression levels (*p* < 0.05).

## Discussion

Growth performance is an indicator reflecting the growth and development of animals. For breeding enterprises, improving the growth performance of animals is of great significance to enhance economic benefits. Among them, F/G is a representative index for evaluating growth performance, and a lower F/G indicates better growth performance (Chen et al., 2022). Based on their common usage in fisheries, BAs have been found to be effective supplements. Specifically, adding 600 mg/kg of BAs to a low fishmeal diet significantly improved the final body weight of *Litopenaeus vannamei* (Li et al., 2023), while a feeding trial using 1.25 g/kg of BAs significantly improved the feed efficiency ratio of *striped catfish* (Adam et al., 2023). Currently, the application of BAs in livestock and poultry is limited. However, it has been found that supplementing BAs can improve the feed efficiency ratio of broiler chickens. Yang et al. (2022) discovered that supplementing 60 mg/kg of BAs could improve the egg-laying performance of laying hens in the later stages. It was also found that supplementing BAs did not affect the production performance of weaned piglets (Chiang and Ferrell, 2020).

The growth performance of animals is closely related to their intestinal morphology (Yousefi et al., 2023; Zahid et al., 2023). The VH, CD and VH/CD are important indicators for measuring intestinal digestion and absorption function (Gu et al., 2022; Martel et al., 2022). BAs have different effects on intestinal cells in various animal species. Supplementation of BAs in the diets of intrauterine growth-restricted piglets can increase the VH/CD of the ileum (Jain et al., 2012). In a mouse model of lipopolysaccharide-induced intestinal injury, adding BAs can improve intestinal cell proliferation and increase VH, thereby protecting the intestinal mucosa. Therefore, BAs supplementation may potentially protect the intestine from injury or infection (Perrone et al., 2010). However, some studies have found that adding BAs to the diet can decrease the VH of the intestine (Parsaie et al., 2007; Liu et al., 2022a,b), which may be related to the concentration and method of use. Furthermore, it is known that BAs participate in lipid metabolism by activating FXR (Wang et al., 1999). This experiment also found that adding BAs can increase the expression levels of FXR in the liver. Studies have shown that FXR plays an important role in the formation and maintenance of the intestinal mucosal barrier. In intestinal epithelial cells, FXR can promote normal intestinal cell proliferation while inhibiting the occurrence of intestinal developmental abnormalities, tumors, and other related diseases. In addition, FXR can also regulate the permeability of the intestinal mucosal barrier and the secretion of the intestinal mucus layer, thereby enhancing the protective function of the intestinal mucosa (Stojancevic et al., 2012).

*ZO-1* is a protein that enhances the stability and integrity of intercellular connections (Gilani et al., 2018). *Claudin-1* and *Occludin* are the main proteins that make up tight junctions between cells. They directly act on the tight connections between epithelial cells and thereby control the permeability of molecules between cells (Song et al., 2018). According to previous studies, dietary supplementation with BAs can increase the abundance of beneficial bacteria and reduce harmful bacteria (Yang et al., 2022). Pathogenic bacteria attach to host cells through adhesion and subsequently use nutrients in host cells to proliferate and release enzymes and toxins, causing damage to the body (Glasser et al., 2001). Some beneficial bacteria have high adhesion capabilities and can inhibit the attachment of pathogenic bacteria to the intestine (Burgain et al., 2013). In this experiment, we observed that the addition of BAs to the diet had no significant impact on jejunum *claudin-1* expression, but enhanced the expression of both *Occludin* and *ZO-1*. Therefore, our findings suggest that supplementing the diet with BAs can significantly improve the F/G of geese during the pre-trial and entire trial period, likely due to the promotion of intestinal health through exogenous BAs supplementation.

Bile acids are synthesized from cholesterol in the liver and stored in the gallbladder in the form of bile salts, which combine with taurine in poultry (Chiang and Ferrell, 2020). When the gallbladder contracts during feeding, it promotes the release of bile salts into the small intestine. The hydroxyl and carboxyl groups of BAs are oriented toward one side as a hydrophilic surface, while the other side consists of an alkyl chain and is hydrophobic, making BAs both hydrophilic and hydrophobic in nature (Li and Chiang, 2014). Lipid is composed of various fatty acids and glycerol molecules, and the interactions between these molecules cause fat to form insoluble droplets that are difficult to break down. Due to its amphiphilic nature, BAs can form a mixture of water and fat, increasing the contact area between lipase and fat, thereby increasing lipolysis (Fuchs, 2003). Additionally, BAs can also enhance the catalytic activity of lipase (Nguyen and Van Do, 2021), leading to the absorption and transportation of fats and fat-soluble microbes (Li and Apte, 2015). Therefore, BAs can help to maintain the steady state of TG and regulate lipid deposition, thereby playing a significant role in lipid metabolism (Hu et al., 2019).

*SREBP-1* is a transcription factor involved in lipid synthesis. It promotes lipid synthesis by regulating the expression of multiple enzymes (Horton et al., 2002). *FAS*, an important enzyme responsible for fatty acid synthesis, plays a crucial role in lipid metabolism by synthesizing triglycerides and other fatty acid esters (Smith, 1994). *SREBP-1* and *FAS* are closely related in lipid metabolism and exhibit positive feedback regulation (Dihingia et al., 2018). *ACCA* is a key limiting enzyme in the fatty acid synthesis pathway. Inhibition of *ACCA* can lead to impaired fatty acid synthesis and stimulation of fatty acid oxidation, thereby altering the rate of fatty acid synthesis (Harwood, 2004). *PPARα* is a transcription factor that can promote lipid metabolism and plays an important regulatory role in the processes of lipid oxidation and *β*-oxidation in the body (Chinetti et al., 2000). Consumption of a high-fat diet can increase the expression of *FAS* and *SREBP-1*, while decreasing the expression of *PPARα* in grouper (Xu et al., 2023). Nevertheless, this expression can be ameliorated by BAs, which can facilitate the breakdown of lipids. Meanwhile, adding a small amount of BAs can reduce the lipid content in the whole body and liver of *Nile tilapia* and *tiger pufferfish* (El-Shenawy et al., 2020; Liao et al., 2020). In this experiment, we found a trend of decreasing expression of liver *SREBP-1* and *FAS* with the addition of BAs in all groups, but there was no significant difference between the groups, which is consistent with previous findings (Liu et al., 2022a,b). However, the expression level of *PPARα* was significantly increased with the addition of 300 mg/kg BAs. Therefore, we believe that the addition of BAs in the diet can promote hepatic lipid metabolism, reduce serum TC and TG levels, and thereby improve abdominal fat deposition in geese.

Bile acids can not only provide feedback inhibition on cholesterol synthesis (Wang et al., 2003), but also enhance the expression of low-density lipoprotein receptor genes on the cell membrane, promoting the absorption and conversion of LDL-C, thereby lowering serum LDL-C levels (Langhi et al., 2008). Furthermore, an increasing number of studies have shown that BAs can also lower serum TG levels (Watanabe et al., 2011). BAs promote the degradation and utilization of TG by regulating the expression of lipid metabolism enzymes in the liver and adipose tissue, thereby lowering serum TG levels. This effect may help reduce animal fat weight, which was also observed in this study.

The gut microbiota contains trillions of microorganisms. The gut microbiota is not only beneficial for extracting nutrients and energy from ingested food, but also produces a large number of metabolites to regulate host metabolism (Ramírez et al., 2017). In this experiment, we analyzed the diversity of each group to determine the preliminary effect of BAs on the gut microbiota of geese. We found that the addition of BAs increased both richness and evenness, which differs from previous studies (Yang et al., 2022). The addition of BAs to the diets of piglets and laying hens did not significantly affect *ɑ*-diversity, which could be due to differences in BAs concentration or different gut segments. However, BAs had a similar effect on *β*-diversity (Liu et al., 2022a,b). The PCoA results showed that the addition of 150 and 300 mg/kg significantly altered the community structure of the gut microbiota. The diversity of gut microbiota is a dynamic process that changes over time. It is generally believed that a high degree of diversity and evenness are signs of mature gut microbiota (Micah et al., 2007).

Bile acids can affect the phylum-level composition of gut microbiota. Similar to our experiment, it has been reported that the supplementation of BAs in rats increased the abundance of Firmicutes and decreased the abundance of Bacteroidetes (Islam et al., 2011; Zheng et al., 2017). A similar trend was also observed in laying hens during the late laying period after supplementation with BAs (Yang et al., 2022). Research has shown a positive correlation between the abundance of Firmicutes and the absorption of energy and nutrients, while an increase in Bacteroidetes may lead to a decrease in nutrient digestibility (Jumpertz et al., 2011). Our study also found that the addition of BAs significantly reduced the abundance of Proteobacteria in the cecum. The acidic nature of BAs may have inhibited the growth of Proteobacteria, which is considered beneficial as bacteria in this phylum can often cause gut inflammation (Mukhopadhya et al., 2012).

The relationship between BAs and gut microbiota is bidirectional. Gut microbiota can affect the metabolism of BAs by modulating the activity of BSH and by influencing a range of enzymes and pathways involved in BAs synthesis (Sun et al.,2023). Most studies have found that *Lactobacillus* and *Bifidobacterium* have higher abundance in the gut of animals supplemented with BAs. This may be due to their strong BSH activity and ability to remove cholesterol, as these bacteria can deconjugate BAs to form free BAs (Fiorucci and Distrutti, 2015; Song et al., 2019). In the present study, dominant genera in the cecum of geese, such as *Bacteroides*, *Prevotella*, and *Faecalibacterium*, were found to produce BSH (Labbé et al., 2014; Song et al., 2019; Burns et al., 2021). We noticed that they are all bacteria that can produce SCFA. Studies have found that there is a mechanism of mutual regulation between SCFA and BAs (Zeng et al., 2019). Through LEfSe analysis, we found that *Blautia* and *Clostridium* were significantly enriched in the 150 mg/kg BAs group. *Blautia* is a type of probiotic bacteria that can participate in carbohydrate metabolism and fatty acid production. Previous study have demonstrated a negative correlation between *Blautia* and visceral fat area (Liu et al., 2021). Additionally, *Clostridium* can utilize BAs as a substrate for growth and replication (Burns et al., 2010). Furthermore, BAs can also inhibit the growth of other competing bacterial populations (Sannasiddappa et al., 2017), which may provide more favorable conditions for the growth of *Clostridium*. According to Chen et al. (2020)‘s research, *Clostridium* are crucial in the cecal metabolism of BAs in mice, resulting in a decrease in the number of other gut bacteria and the amount of BAs in feces. Furthermore, our study found that supplementing BAs in the cecum resulted in a significant change in the amount of SCFA in the gut, This is correlated with the total concentration of SCFA detected in the cecum and jejunum. It is generally recognized that a higher concentration of SCFA is beneficial for animals, as they play a crucial role in maintaining intestinal mucosal health, regulating intestinal pH, modulating energy metabolism by promoting glucose metabolism and fatty acid oxidation (Den et al., 2013), and enhancing intestinal immunity (Xu et al., 2020). These findings suggest that the relationship between BAs, intestinal microbiota, and SCFA is bidirectional, and that BAs can elevate SCFA levels, thereby promoting the health of geese.

In summary, dietary supplementation with 150 mg/kg BAs was found to reduce the F/G in Holdobagy geese at 35–63 days. BAs, as an effective dietary additive, can improve lipid metabolism and gut health of geese by improving intestinal morphology, mucosal barrier, intestinal SCFA concentration and change the microbial community structure in the cecum.

## Data availability statement

The datasets presented in this study can be found in online repositories. The names of the repository/repositories and accession number(s) can be found in the article/supplementary material.

## Ethics statement

The animal study was reviewed and approved by Laboratory Animal Ethics Committee of the Shanghai Academy of Agricultural Sciences.

## Author contributions

GL and XW contributed equally to this study, covering conceptualization, methodology, investigation, and original draft preparation. YL, SG, YY, and CW assisted with data collecting, formal analysis, and review and editing. HW and DH co-supervised the project and provided the funding. All authors have read and approved the final manuscript.

## Funding

This research was funded by the Climbing Plan of Shanghai Academy of Agricultural Sciences (PG21171), the SAAS Program for Excellent Research Team (2022–021), and the China Agriculture Research System of MOF and MARA (CARS-42-35).

## Conflict of interest

The authors declare that the research was conducted in the absence of any commercial or financial relationships that could be construed as a potential conflict of interest.

## Publisher’s note

All claims expressed in this article are solely those of the authors and do not necessarily represent those of their affiliated organizations, or those of the publisher, the editors and the reviewers. Any product that may be evaluated in this article, or claim that may be made by its manufacturer, is not guaranteed or endorsed by the publisher.
